# Early use of levosimendan to improve survival and neurological outcomes after cardiac arrest with myocardial dysfunction: a protocol for a randomized controlled trial

**DOI:** 10.3389/fcvm.2026.1850394

**Published:** 2026-09-17

**Authors:** Zhen De, Nengyuan Xu, Hua Zhang, Fan Yang, Lanfang Du

**Affiliations:** 1 Department of Emergency Medicine, Peking University Third Hospital, Beijing, China; 2 Clinical Epidemiology Research Center, Peking University Third Hospital, Beijing, China

**Keywords:** cardiac arrest, levosimendan, myocardial dysfunction, post-cardiac arrest syndrome, randomized controlled trial

## Abstract

**Introduction:**

Post-cardiac arrest myocardial dysfunction (PAMD) is a major contributor to early death (within 72 hours) following return of spontaneous circulation (ROSC). Levosimendan, a calcium-sensitizing inotrope with vasodilatory and mitochondrial-protective properties, offers a potential therapeutic strategy by increasing cardiac output, improving organ perfusion, and attenuating mitochondrial ischemia-reperfusion injury. While experimental evidence suggests multi-organ benefits in cardiac arrest (CA), robust clinical data are lacking.

**Methods and analysis:**

This exploratory, partially blinded, randomized, placebo-controlled trial will enroll 60 comatose CA patients with PAMD across three centers in China. Within 180 minutes after ROSC, eligible participants will be randomized (1:1) to receive either intravenous levosimendan (0.05–0.2 μg/kg/min for 24 hours) or volume-matched saline placebo; the assigned infusion will begin within 6 hours after ROSC. The primary outcome is 30-day all-cause mortality, interpreted within an exploratory signal-generating framework. Secondary outcomes explore 30-day neurological recovery assessed by the Cerebral Performance Category (CPC) and Modified Rankin Scale (mRS), along with cardiac function and hemodynamic stability during the first week post-resuscitation.

**Discussion:**

This exploratory trial is designed to align early levosimendan infusion (within 6 hours post-ROSC) with its pharmacokinetics to cover the anticipated nadir of myocardial dysfunction. By targeting a homogeneous cohort with witnessed arrest, rapid ROSC (<60 min), and LVEF <40%, the trial focuses on patients with potentially reversible injury who may benefit most. The findings will provide preliminary evidence on efficacy and safety to inform a definitive Phase III trial.

**Clinical Trial Registration:**

https://clinicaltrials.gov/study/NCT05956431, ClinicalTrials.gov NCT05956431. Registered on 21 July 2023.

## Strengths and limitations of this study

**Strengths:** The main strengths of this study include its design as one of the first randomized controlled trials evaluating levosimendan in this specific population, the rigorous multicenter, partially blinded, placebo-controlled methodology with centralized randomization, and the implementation of standardized post-resuscitation care to minimize co-intervention bias.

**Limitations:** Several limitations must be considered when interpreting the results of this exploratory trial. First, as an exploratory study, the sample size is not powered to detect moderate or small clinical benefits; a non-significant result must be interpreted with caution as it may reflect either a true lack of efficacy or inadequate statistical power. Second, the strict inclusion criteria (witnessed OHCA, ROSC <60 minutes, LVEF <40%, enrollment within 3 hours) define a specific subgroup of post-cardiac-arrest patients, and findings may not extend to unwitnessed arrests, in-hospital cardiac arrests, or patients with preserved cardiac function. Third, the trial includes multiple secondary and safety endpoints without adjustment for multiple comparisons; therefore, these findings are considered hypothesis-generating and require confirmation in future studies. Fourth, while participants and outcome assessors are blinded, the treating clinical team is not due to the need for hemodynamic management, which could theoretically influence clinical decisions despite protocolized care. Fifth, conducted in tertiary hospitals in Beijing, China, the results may not be fully generalizable to other healthcare systems. Finally, since the comparator is placebo rather than an active inotrope, a positive result cannot differentiate levosimendan-specific pleiotropic effects from the nonspecific benefits of any early inotropic support.

**Interpretation Framework:** Given these considerations, the results of this trial will be interpreted primarily as providing preliminary evidence and robust effect size estimates to guide the design of any subsequent definitive, large-scale clinical trial.

## Open science

### Where the trial protocol and statistical analysis plan can be accessed {5}

The full protocol and statistical analysis plan (SAP) for this trial will be made accessible as Supplementary Materials alongside the primary publication in a peer-reviewed journal.

### Plans to communicate trial results {8}

Trial results will be disseminated through multiple channels regardless of outcome. The primary results will be submitted for publication in a peer-reviewed international journal within 12 months of trial completion, following the International Committee of Medical Journal Editors (ICMJE) guidelines for authorship. Results will also be presented at relevant scientific conferences and uploaded to the clinical trial registry (ClinicalTrials.gov NCT05956431). All participating patients (or their legal representatives) will receive a lay-language summary of the main findings upon request. The full protocol and statistical analysis plan will be made publicly available as Supplementary Materials to the primary publication.

## Introduction

### Background and rationale {9a}

Cardiac arrest (CA) is one of the leading causes of death in the world with low survival rates ranging between 1.2% (1) and 9.1% (2). Successful resuscitation often triggers post-cardiac arrest syndrome (PCAS), a life-threatening multiorgan failure syndrome characterized by (1) post-cardiac arrest brain injury; (2) post-cardiac arrest myocardial dysfunction; (3) systemic ischemia/reperfusion response; (4) persistent precipitating pathology (3). Post-cardiac arrest myocardial dysfunction (PAMD) manifests as impaired systolic and/or diastolic function, resulting in compromised cardiac output and systemic hypoperfusion (4). Yao et al. (5) found that among 190 patients who successfully achieved return of spontaneous circulation (ROSC) after out-of-hospital cardiac arrest (OHCA), nearly half (44%) exhibited early myocardial dysfunction [left ventricular ejection fraction (LVEF) < 40%], indicating a high incidence of PAMD, which is relatively common in post-cardiac arrest patients and has been identified as a predominant cause of early death within 72 hours post-resuscitation (6).

### Pathophysiological basis of PAMD and pharmacological rationale for levosimendan

In clinical practice, PAMD may respond to low-dose inotropic support. Its natural course is often transient: cardiac index may reach a nadir approximately 8 hours after ROSC, show marked recovery by 24 hours, and approach normal by 72 hours (3, 38).

The pathophysiology of PAMD involves several key mechanisms (4, 6, 7). Firstly, ischemia-reperfusion injury (IRI) is central to PAMD pathogenesis. Ischemia impairs mitochondrial function, depleting cellular energy and causing Na⁺/K⁺-ATPase failure, which leads to sodium accumulation, cellular edema, and increased ventricular stiffness. Subsequent calcium overload via Na⁺/Ca^2^⁺ exchange activates damaging enzymes, impairing contractility and increasing oxygen demand. Reperfusion then generates excessive reactive oxygen species (ROS), causing secondary injury, inactivating metabolic enzymes, and further depleting energy; resultant inorganic phosphate accumulation and acidosis collectively reduce myofilament Ca^2^⁺ sensitivity (8). Secondly, inflammatory cytokines (e.g., TNF-*α*, IL-6) mediate systemic I/R injury following ROSC. These cytokines trigger a systemic inflammatory response syndrome (SIRS)-like response that directly impairs myocardial function and induces pathological vasodilation, thereby compromising hemodynamics. Thirdly, excess catecholamines exacerbate calcium overload and ROS production, worsening myocardial dysfunction. Finally, microcirculatory dysfunction perpetuates ischemic injury. Post-reperfusion coagulation activation promotes microthrombosis. Concurrently, neutrophil and platelet aggregation causes “no-reflow,” while endothelial swelling and luminal narrowing further obstruct perfusion.

Given the reversible nature of PAMD, the administration of inotropic agents during cardiac dysfunction may theoretically improve clinical outcomes in CA patients. Levosimendan is a calcium sensitizer whose primary mechanism involves enhancing the sensitivity of cardiac troponin C to calcium ions. This directly increases myocardial contractility without elevating intracellular calcium concentration, thereby improving cardiac function while avoiding calcium overload-associated arrhythmias and increased myocardial oxygen consumption. Concurrently, levosimendan activates ATP-sensitive potassium (K_ATP_) channels on vascular smooth muscle cell membranes, eliciting vasodilation and improving organ perfusion. Furthermore, levosimendan activates K_ATP_ channels on the mitochondrial inner membrane, preventing mitochondrial matrix contraction and intermembrane space expansion which limits cellular respiration under ischemic conditions (9). Additionally, its anti-inflammatory properties—reducing pro-inflammatory cytokines (IL-6, IL-8), adhesion molecules, and ROS—further ameliorate systemic inflammation integral to PCAS and PAMD (10, 11).

### Pre-clinical data on efficacy of levosimendan

Preclinical studies demonstrate that levosimendan provides multi-organ protection following cardiac arrest. Its core mechanism involves rapidly enhancing cardiac function, stabilizing hemodynamics, and increasing cardiac output and systemic perfusion (12). This improvement directly elevates cerebral blood flow, reduces neuronal injury, and significantly improves neurological outcomes early post-resuscitation (e.g., within 24 hours) (13–15); it also effectively alleviates hypoperfusion in peripheral organs (e.g., the kidneys (16, 17), lungs (18)). Levosimendan further exerts direct protection through multiple independent mechanisms (10, 11): it independently improves cerebral tissue oxygenation within 30 minutes post-arrest by directly optimizing mitochondrial function via mitoK_ATP_ channel activation (19); while also improving intestinal microcirculation dysfunction partly independent of overall blood flow, enhancing mesenteric blood supply and tissue oxygenation (20); and mitigates systemic inflammation, inhibits apoptosis, and regulates protective pathways (e.g., ERK activation) to reduce ischemia-reperfusion injury in kidneys (21, 22) and other organs. Collectively, these preclinical findings suggest that levosimendan may confer multi-organ protection after cardiac arrest through a dual mechanism of improving systemic hemodynamics and direct cellular effects. Whether these promising experimental benefits translate into improved clinical outcomes in human patients remains an open question that this trial aims to address.

### Clinical trials in humans

Clinical evidence specifically evaluating levosimendan in cardiac-arrest patients remains limited. In a retrospective observational study of 940 OHCA patients, levosimendan use within 24 hours after ICU admission was not associated with 30-day mortality after adjustment. In an exploratory within-treatment timing analysis, initiation 0-6 hours after ICU admission was associated with lower adjusted 30-day mortality than later initiation; this hypothesis-generating finding may be affected by residual confounding (23). Further randomized trials are required to assess efficacy and optimal timing in this population.

Based on this evidence, we designed a multicenter randomized controlled trial (RCT) to determine whether levosimendan administered within 6 h post-resuscitation improves clinical outcomes in comatose CA patients with low cardiac output.

### Explanation for the choice of comparators {9b}

Placebo (0.9% saline) was selected as the comparator to isolate the specific effects of levosimendan, with saline matching the drug solution in volume and appearance. This approach is justified by the absence of proven targeted therapies for post-cardiac arrest myocardial dysfunction in resuscitated OHCA patients. Thus, placebo comparison is mandated to isolate drug effects from unavoidable variations in standard care.

## Objectives {10}

### Primary objectives

This exploratory study aims to assess the safety and preliminary signal of levosimendan on 30-day mortality post-ROSC in comatose resuscitated OHCA patients and to provide feasibility, effect-estimation, and protocol-adherence data for the design of a future definitive trial.

### Secondary objectives

The secondary objectives are to explore the potential effects of levosimendan on: (1) cardiac function and hemodynamic stability during the first week post-resuscitation; and (2) neurological recovery assessed by the Cerebral Performance Category (CPC) and Modified Rankin Scale (mRS) at 30 days post-ROSC. Specific measurement methods are detailed in the “Outcomes{16}” section.

## Methods: patient and public involvement, trial design

### Trial design {12}

The study was designed as a multicenter, partially blinded, 1:1 randomized, placebo-controlled exploratory trial. The study protocol was approved by the Institutional Ethics Committee of Peking University Third Hospital (Approval No. 331-02) and registered at ClinicalTrials.gov (NCT05956431) prior to participant enrollment. No participant has been enrolled to date; trial registration and ethical approval were completed before participant screening and enrollment procedures commenced. The trial sponsor is Peking University Third Hospital, Beijing, China. Sponsor contact: Lanfang Du (angelslovedudu@126.com). The sponsor provided infrastructural support but had no role in study design, data collection, management, analysis, interpretation, or the decision to submit the report for publication. The funders named under ‘Funding’ had no role in any aspect of the trial.

### Patient and public involvement {11}

Patients or the public were not involved in the design, conduct, reporting, or dissemination plans of this research. The acute and critical nature of the condition under study (post-cardiac arrest coma) meant that involving patients in the initial design phase was not feasible. However, the results of the trial will be disseminated to the public through a lay-language summary, which will be made available to participants (or their legal representatives) upon request.

## Participants, interventions, and outcomes

### Study setting {13}

The trial will be conducted in the emergency departments and intensive care units of three tertiary academic hospitals in China:

1. Peking University Third Hospital
2. Peking University First Hospital
3. Xuanwu Hospital

Enrollment period: May 2026 – December 2027

### Eligibility criteria {14a}

Comatose adult patients admitted to the participating ICUs following successful resuscitation from out-of-hospital cardiac arrest (OHCA) will be consecutively screened for eligibility. Screening is performed by the site investigator at each center. The REDCap electronic data capture system will serve as the centralized screening log throughout the trial. Eligible patients will be randomized and the study intervention initiated within the pre-specified 6-hour window post-ROSC. Participation in this trial does not preclude enrollment in other non-interventional clinical studies.

### Eligibility criteria for sites and for individuals who will deliver the interventions {14b}

The trial will be conducted in the emergency departments and intensive care units of three tertiary academic hospitals in China, selected for their extensive experience in post-resuscitation care and high annual volume of cardiac arrest admissions. The study intervention will be administered by trained physicians and critical care nurses within the participating ICUs who have completed the mandatory trial-specific training.

### Inclusion criteria

1. Age ≥18 years
2. Witnessed OHCA with successful ROSC
3. Time from arrest to ROSC < 60 minutes
4. PAMD, confirmed by LVEF <40% on transthoracic echocardiography performed before randomization and within 180 minutes after ROSC. To minimize inter-site variability, all participating centers will follow a common echocardiographic acquisition and measurement protocol. Whenever technically feasible, apical four- and two-chamber views will be obtained and LVEF will be calculated using the biplane method of disks (modified Simpson method), as recommended by the American Society of Echocardiography and the European Association of Cardiovascular Imaging (24). Site echocardiographers will undergo protocol-specific training and calibration before participation. For an initial LVEF of 35%-45%, classification uncertainty around the 40% eligibility threshold, or technically limited images, a second qualified senior echocardiographer will independently review the study before randomization. A between-reader difference >5 percentage points or disagreement regarding classification as <40% versus ≥40% will trigger consensus review; if eligibility cannot be resolved within the 180-minute enrollment window, the patient will not be randomized. All baseline images will be archived and will undergo blinded secondary re-reading before database lock by a qualified echocardiographer who was not involved in treatment decisions. Inter-rater reliability will be summarized using an absolute-agreement intraclass correlation coefficient (ICC) for continuous LVEF and Cohen's kappa for the binary <40% eligibility classification.
5. Comatose state post-ROSC [Glasgow Coma Scale (GCS) score < 8]
6. Completion of enrollment procedures (screening, consent, randomization) within 180 minutes post-ROSC

### Exclusion criteria

1. Extracorporeal cardiopulmonary resuscitation (ECPR) use
2. Pre-arrest CPC≥3 or conditions confounding neurological outcome assessment
3. Estimated creatinine clearance (eCrCl) < 30 mL/min or pre-arrest renal replacement therapy (RRT)
4. Pregnancy (confirmed or suspected)
5. Intracranial hemorrhage (as arrest etiology or complication)
6. Terminal illness
7. Refuse to participate

### Withdrawal criteria

1. Study drug-related serious adverse event (SAE) prompting infusion discontinuation for safety
2. Consent withdrawal by Legally Authorized Representative (LAR) or patient regaining decision-making capacity
3. Loss to follow-up before day-30 endpoint

### Interventions {15a}

#### Intervention description

In the levosimendan group, patients receive continuous intravenous levosimendan infusion at 0.05–0.2 μg/kg/min (no bolus administered) initiated within 6 hours post-ROSC and maintained for 24 hours. The infusion solution is prepared by diluting 12.5 mg levosimendan concentrate in 5% glucose. All patients concurrently receive standardized evidence-based PCAS bundle care, including targeted temperature management, hemodynamic optimization (MAP >65 mmHg), mechanical ventilation protocol, glycemic control (blood glucose 7.8-10 mmol/L) and institutional ICU supportive care. Concomitant use of other inotropic agents (e.g., dobutamine) is prohibited during the 24-hour infusion period; vasopressors (preferably norepinephrine) are permitted for hypotension management.

In the placebo group, patients receive identical intravenous infusion of 0.9% saline matching levosimendan in volume, infusion rate (weight-adjusted), and duration (24 hours), initiated within 6 hours post-ROSC. Identical PCAS bundle care and concomitant medication restrictions apply.

### Criteria for discontinuing or modifying allocated interventions {15b}

If the legal surrogate (prioritized as spouse > parents > adult children per Civil Code) or Ethics Committee-appointed proxy withdraws consent, the patient shall be immediately withdrawn from the trial without compromising clinical care; should the patient regain decision-making capacity and refuse continued participation, all study procedures cease upon refusal documentation. A participant may be withdrawn by the investigator upon Ethics Committee approval if life-threatening intercurrent illness unrelated to cardiac arrest emerges, the competent patient explicitly refuses further observations, or continued enrollment is medically detrimental as jointly determined by the investigator and an independent intensivist. For all withdrawals, endpoint assessments shall be completed through the exit date with documented rationale using China National Medical Products Administration (NMPA) Form 12, and any adverse events followed until resolution or stabilization per Good Clinical Practice guidelines.

### Strategies to improve adherence to interventions {15c}

All participating physicians and study coordinators possess relevant critical care and cardiology expertise. They will complete mandatory training, which includes an instructional video, review of the study protocol, and reference materials (protocol summaries and pocket cards). The study intervention is administered as a continuous 24-hour infusion in the ICU, following a standardized protocol. The fixed nature of the infusion regimen and its delivery in a controlled ICU environment minimize the risk of administration delay or omission. All clinical staff involved in the patient's care during hospitalization and those responsible for follow-up assessments are trained on study-specific procedures to ensure consistent protocol adherence, management, and data collection.

### Relevant concomitant care permitted or prohibited during the trial {15d}

All standard supportive care for post-cardiac arrest patients is permitted, including mechanical ventilation, vasopressors (norepinephrine preferred), antiarrhythmics, antibiotics as clinically indicated, antiepileptics, renal replacement therapy, and nutritional support; however, use of other positive inotropic agents (e.g., dobutamine, milrinone, epinephrine for inotropy) is prohibited during the 24-hour study infusion period to avoid confounding, while norepinephrine for vasopressor support remains permissible. Post-infusion, clinicians may utilize any necessary medications. Protocol deviations—including alteration of the assigned infusion regimen or premature discontinuation without meeting predefined criteria {15b}, or non-justified modification of standardized PCAS bundle care—are strongly discouraged except for safety or awakening concerns.

### Outcomes {16}

#### Mortality at 30 days

All-cause mortality at 30 days post-ROSC (Day 0 = day of ROSC) will be ascertained through hospital record review and/or direct contact (telephone or in-person visit) with the patient or their legally authorized representative.

### Secondary outcomes

#### Cardiac function, hemodynamic parameters and neurological recovery

Cardiac function will be assessed through serial measurements: cardiac output will be monitored hourly for the first 6 hours and every 6 hours until 72 hours post-ROSC using the PiCCO hemodynamic monitoring system; transthoracic echocardiography will be performed at 24 hours, 72 hours, and 1 week post-ROSC; and the myocardial injury biomarker NT-proBNP will be measured at 12 hours, 24 hours, 48 hours, 72 hours, and 1 week post-ROSC. Hemodynamic parameters, including systolic and mean arterial pressure, will be monitored hourly until 1 week post-ROSC. Neurological recovery will be assessed by the CPC and mRS at 30 days post-ROSC.

#### Safety

Cumulative daily incidence of adverse events (AEs) during the first 7 days, including serious adverse events (SAEs) and suspected unexpected serious adverse reactions (SUSARs).

#### Harms {17}

Adverse events (AEs) will be monitored and recorded continuously throughout the trial period. Specific attention will be paid to known levosimendan-associated adverse effects, including hypotension, tachycardia, and arrhythmias. All patients will undergo continuous hemodynamic monitoring (blood pressure, heart rate, cardiac rhythm) during the 24-hour infusion period and for at least 7 days post-infusion or until clinically stable. All AEs will be assessed daily for the first 7 days and at all follow-up visits. Relationship to study intervention will be categorized as probable, possible, unlikely, or unknown.

### Participant timeline {18}

Besides the section presenting outcomes above ({16}), a flow chart for the trial is presented in Figure 1, and a time schedule for the trial is presented as a schematic diagram in Table 1.

**Figure 1:**
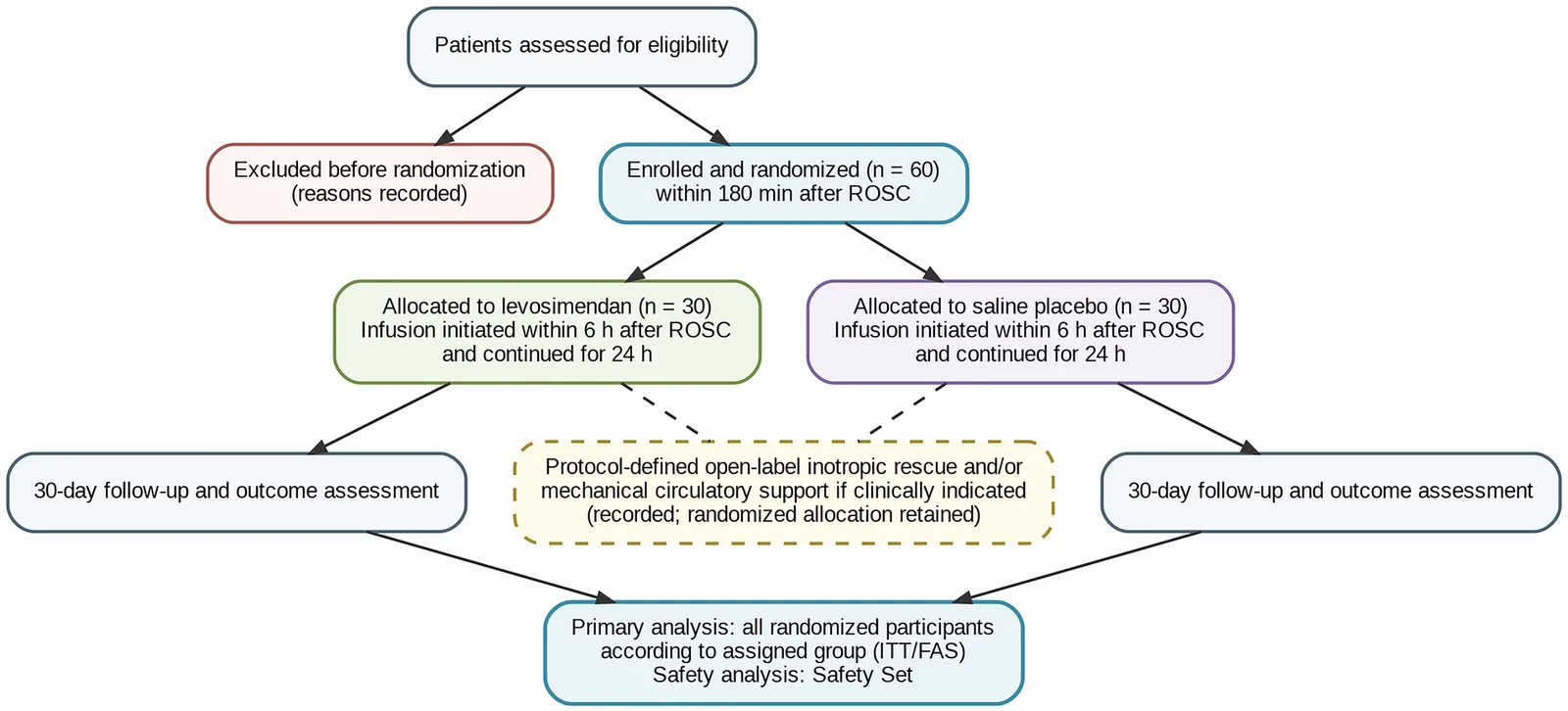
Trial flow chart.

**Table 1:** Time schedule for the trial.

Timepoint	Trial Period							
	Enrollment	Post-randomization						Follow-up
	≤3 h	0–24 h (start ≤6 h)	12 h	24 h	48 h	72 h	1 week	30 days
Enrollment								
Eligibility screening	✓							
Surrogate informed consent^a^	✓							
Baseline data and TTE^b^	✓							
Randomization	✓							
Interventions								
Levosimendan infusion		✓						
Saline placebo infusion		✓						
Infusion adjustment/discontinuation		✓						
Rescue/Concomitant Care								
Open-label rescue inotrope		✓						
Rescue MCS		✓	✓	✓	✓	✓	✓	
Assessments								
SBP and MAP	✓	✓	✓	✓	✓	✓	✓	
Cardiac output (PiCCO)	✓	✓	✓	✓	✓	✓		
TTE	✓			✓		✓	✓	
NT-proBNP			✓	✓	✓	✓	✓	
Renal function^c^			✓	✓	✓	✓	✓	
NSE			✓	✓	✓	✓	✓	
Head CT (if clinically indicated)				✓		✓	✓	
AEs and SAEs		✓	✓	✓	✓	✓	✓	✓
CPC score								✓
mRS score								✓
Survival status						✓	✓	✓

ROSC, return of spontaneous circulation; SBP, systolic blood pressure; MAP, mean arterial pressure; CO, cardiac output; TTE, transthoracic echocardiography; NSE, neuron-specific enolase; CT, computed tomography; CPC, cerebral performance category; mRS, modified Rankin Scale.

a Informed consent obtained from legal surrogate (prioritized as spouse > parents > adult children per Civil Code).

b Baseline data include demographic and pre-ROSC clinical characteristics.

c Serum creatinine and eGFR.

### Sample size {19}

This is an exploratory trial primarily aimed at estimating the treatment effect and safety profile of levosimendan, rather than definitively testing for superiority. Consequently, the sample size is based on recruitment feasibility and precision considerations rather than formal statistical power. The target of 60 participants (30 per group) was determined by estimated recruitment capacity across the participating centers over the planned study period. With this sample size, the study will provide effect estimates with acceptable precision for the primary outcome; for example, assuming a control-group 30-day mortality of 60%, a sample of 30 patients per group would yield a 95% confidence interval for the risk difference with a half-width of approximately 25 percentage points around the observed effect. While this precision is insufficient for confirming modest treatment effects, it provides useful point and interval estimates—including observed effect size, variability, recruitment, and protocol adherence—to inform the design and sample size calculation of a future definitive Phase III trial.

### Recruitment {20}

Recruitment is planned over a 20-month active screening period from May 2026 to December 2027 across the three participating tertiary hospitals. Although ethics approval was obtained in June 2023, study initiation was delayed by funding-allocation procedures, investigational-drug procurement and quality testing, and sequential ethics requirements at the sub-centers. The coordinating center began active screening in May 2026. From May to July 2026, four patients were screened; one met the stringent eligibility criteria but the legal representative declined participation, and no participant had been randomized at the time of this revision. Recruitment will be reviewed monthly using the REDCap screening log. If enrollment remains below projection, contingency measures include strengthening pre-hospital-to-hospital coordination and early eligibility pre-assessment, standardized training of research coordinators for timely family communication while fully respecting voluntary consent, accelerating activation of the participating sub-centers to enable parallel screening, and evaluating additional referral or participating centers subject to ethics and protocol approval. These measures are intended both to support recruitment and to reduce temporal drift and protocol fatigue through repeated staff training and centralized monitoring.

## Assignment of interventions: allocation

### Sequence generation {21a} {21b}

The allocation sequence will be generated by an independent biostatistician using SAS software (version 9.4), with a 1:1 randomization employing randomly permuted blocks of sizes 4 and 6. To ensure allocation concealment, this sequence will be implemented and maintained within the REDCap electronic data capture system (version 13.5). Randomization will be stratified by the three participating clinical sites (Peking University Third Hospital, Peking University First Hospital, and Xuanwu Hospital) to ensure balance across centers. After a patient is successfully screened and formally enrolled, the treating physician will log into the secure REDCap portal, input the patient's study ID and basic demographics, and the system will reveal the group assignment (levosimendan or placebo) in real time. Thus, the allocation sequence remains concealed until enrollment, whereas designated personnel involved in study-drug management are unblinded after randomization. Outcome assessors and data analysts remain blinded. Emergency unblinding for other blinded clinical personnel will be permissible only in exceptional clinical circumstances through a dedicated secure REDCap function, with the event immediately reported to the principal investigator and steering committee.

### Concealment mechanism {22}

Allocation concealment is strictly maintained through the REDCap system. The random allocation sequence is stored and hidden within REDCap. After a patient is confirmed to be eligible and written informed consent is obtained, the treating physician logs into the REDCap system and enters the patient's data. Only at this point does the system automatically assign and display the treatment group (Levosimendan or placebo). This ensures that the treatment assignment for the next patient is always hidden until after the current patient is formally enrolled, preventing selection bias.

### Implementation {23}

The allocation sequence will be generated by an independent clinical statistics expert using SAS software (v9.4). Participant enrolment will be conducted by the site investigators at each clinical center, who are responsible for confirming patient eligibility and obtaining informed consent. Following enrolment, the treatment assignment is automatically executed by the central REDCap system (v13.5), which reveals the group allocation to the investigator. Subsequently, an unblinded pharmacist at each site, who is not involved in any other trial procedures, will prepare the corresponding numbered study kit (Levosimendan or placebo) based on the assignment provided by REDCap for administration to the patient.

## Assignment of interventions: blinding

### Who will be blinded {24a}

This trial employs a partially blinded design. Participants (who are unconscious during the critical intervention period), outcome assessors (e.g., for the Day 30 Cerebral Performance Category score), and data analysts are blinded to the treatment assignment. Bedside clinical staff involved in drug administration remain unblinded due to the requirements of treatment management. To minimize performance bias, unblinded clinicians are required to strictly adhere to standardized, protocol-driven guidelines for vasoactive support, fluid administration, and prognostication.

### How blinding will be achieved {24b}

Although bedside clinicians are unblinded to the treatment, the investigational product (Levosimendan) and the saline placebo are prepared by the unblinded hospital pharmacy to be identical in appearance (both are colorless solutions), volume, and packaging. All infusion bags are labeled identically with only the patient study ID and “Study Drug Infusion” to maintain blinding for the participants, outcome assessors, and other clinical staff not responsible for drug administration. The pharmacy maintains the master randomization list, which is securely stored separately from all clinical data. The unblinding of the treatment allocation for the entire study will only occur after the database has been locked and all primary analyses are completed.

### Procedure for unblinding if needed {24c}

Emergency unblinding is strictly reserved for life-threatening emergencies in which knowledge of the study drug is necessary for clinical management and the designated unblinded study personnel are not available. In such rare scenarios, the covering physician may reveal the treatment assignment through a secure REDCap module using secured credentials; the system will immediately notify the site PI. All instances will be comprehensively documented (rationale and time) in the TMF and eCRF and reported to the sponsor-investigator and ethics committee within 24 hours. Emergency unblinding will not alter the randomized-group assignment; all available efficacy and safety outcomes will continue to be collected and the participant will remain in the intention-to-treat analysis unless consent for further follow-up is withdrawn.

### Standardization of post-cardiac arrest care

To minimize treatment heterogeneity and potential confounding, all participating centers must adhere to standardized post-arrest care pathways. Targeted temperature management (TTM) is maintained at a target of 32-36°C for at least 24 hours, followed by controlled rewarming of 0.1-0.2 ℃/h. Sedation and analgesia are protocolized to achieve a target RASS of −4 to −5 during the intervention period, with systematic daily interruption only after TTM completion. Neuroprognostication strictly follows a delayed multimodal approach: assessments are deferred until at least 72 hours post-ROSC (and 24 hours post-rewarming), and only after excluding potential confounders like residual sedation. Prognostication integrates clinical examination, brain imaging, serum biomarkers (NSE), and neurophysiology (EEG and bilateral N20 SSEPs), with a poor prognosis declared only when concordant findings across ≥2 modalities indicate near-zero probability of recovery. Furthermore, all withdrawal-of-life-sustaining-therapy (WLST) decisions are made collectively by a dedicated Cardiac Arrest Multidisciplinary Team (CAMDT) instead of by individual clinicians, based on these predefined objective criteria, and finalized through shared decision-making with the patient's family.

Non-ECPR mechanical circulatory support (MCS), including intra-aortic balloon pump (IABP), percutaneous ventricular assist devices (Impella), or VA-ECMO initiated after ROSC, is permitted post-randomization when clinically indicated at the discretion of the treating team. All MCS details, including device type, clinical indication, initiation and weaning timestamps, duration, and associated hemodynamic context, will be meticulously recorded in the eCRF. MCS use will not alter randomized treatment assignment or exclusion from the primary ITT/FAS analysis.

### Open-label inotropic rescue protocol

To address patient safety concerns regarding refractory low-output failure, predefined open-label rescue criteria are established. Inotropic rescue is mandated if a patient develops refractory tissue hypoperfusion, defined as persistent lactate ≥6 mmol/L or a rise >2 mmol/L over 2 consecutive measurements despite adequate volume status and norepinephrine support, accompanied by echocardiographic evidence of worsening contractility (LVEF <30% or VTi <10 cm). Dobutamine is the preferred rescue agent. The study blind is not broken at the time of rescue initiation, and all medication details are meticulously recorded in the eCRF.

## Data collection and management

### Plans for assessment and collection of outcomes {25a}

All study data, including eligibility screening, consent, baseline characteristics, intervention details, serial measurements, outcome assessments, and adverse events, will be prospectively collected using electronic case report forms (eCRFs) hosted on the REDCap platform (version 13.5). Source data will be extracted directly from patient medical records, laboratory and hemodynamic monitoring systems, and imaging reports. The 30-day all-cause mortality endpoint will be ascertained through medical record review or direct contact. Neurological outcome, assessed by the CPC and mRS scores at day 30, will be determined by two independent neurologists who remain blinded to treatment allocation. Prior to study initiation, both assessors will undergo standardized training and inter-rater calibration exercises to ensure consistent application of the CPC and mRS scales. The 30-day neurological assessment will be conducted via a structured telephone interview using a standardized script to ensure consistency. In the event of inter-rater disagreement, the two neurologists will discuss the case to reach a consensus; if consensus cannot be achieved, a third senior neurologist (also blinded to allocation) will adjudicate the final CPC and mRS scores.

Biological specimens will be collected at predefined time points (24 h, 48 h, 72 h, and 1 week post-ROSC) for protocol-specified laboratory testing. All samples are processed immediately for analysis of myocardial injury, neurological damage, renal function, and inflammatory biomarkers. The remaining aliquots will be stored in a dedicated research biobank at −80°C for potential future validation studies, subject to approval by the relevant ethics committee and regulatory authorities. The results of all laboratory tests will be retained in the study database.

### Plans to promote participant retention and complete follow-up {25b}

Given the critical nature of the condition and the 30-day primary endpoints, strategies are implemented to ensure complete follow-up. Throughout the index hospitalization, the research team will maintain close communication with the patient's legally authorized representative (LAR) to emphasize the importance of the study. To maximize retention for the day 30 assessment, a flexible approach is adopted, offering the blinded outcome evaluation via telephone, video call, or an in-person visit based on participant preference and circumstance. Systematic reminders (phone call, SMS) will be sent prior to the follow-up window. All contact attempts and reasons for any loss to follow-up will be meticulously documented.

### Data management {26}

All study data will be securely managed using the REDCap electronic data capture system. Data quality is safeguarded through automated validation checks during entry, systematic queries for inconsistencies, and source data verification for all primary outcomes. The system maintains a complete audit trail and employs role-based access controls to ensure data integrity and confidentiality throughout the trial.

## Statistical methods

### Statistical methods for primary and secondary outcomes {27a} {27b} {27c} {27d}

Statistical analyses will be conducted using R software (version 4.x) by epidemiologists who will remain blinded to treatment allocation. Consistent with the exploratory design, the primary analysis will be performed on the Full Analysis Set (FAS) adhering to intention-to-treat (ITT) principles. The Safety Set will be used for safety evaluations. To address the potential confounding effect of rescue therapy and mechanical circulatory support (MCS) use—recognizing their potential role as severity markers rather than treatment effect modifiers—both Per-Protocol Set (PPS) and As-Treated (AT) sensitivity analyses will be conducted alongside the primary ITT analysis. The PPS analysis will restrict the cohort to participants who adhered to the protocol without receiving non-protocolized rescue therapies, thereby estimating biological efficacy under optimal adherence. The AT analysis will reclassify participants based on the actual treatment received, including those crossing over to rescue therapies, to explicitly evaluate how rescue therapy influences the treatment effect estimate. Where initiation times differ meaningfully between groups and event counts are sufficient to support model estimation, an exploratory sensitivity analysis treating rescue therapy and MCS initiation as time-dependent covariates will be performed; otherwise, these post-randomization events will be summarized without forcing a time-dependent model. The incidence of rescue therapy and MCS use between the two groups will also be reported (all MCS details, including device type and timestamps, are meticulously recorded in the eCRF).

Consistent with the exploratory design, analyses will emphasize effect estimation (95% CIs) over formal hypothesis testing. For the primary outcome (30-day mortality), risk difference and relative risk with 95% CIs will be reported. Additionally, Kaplan–Meier curves will be generated to visually describe survival trajectories, and univariable Cox regression will be used to report the hazard ratio; multivariable Cox regression will not be performed due to the limited sample size and the exploratory nature of the trial. Descriptive P-values may be provided but are not confirmatory. For interpretability, absolute risk reduction (ARR) will be defined as the 30-day mortality risk in the placebo group minus that in the levosimendan group, such that positive values favor levosimendan. To guide consideration of a future Phase III trial, we pre-specify a pragmatic, non-binding progression framework informed by target effect sizes used in contemporary post-cardiac-arrest trial designs (25–27). A point estimate of ARR ≥6% (approximately RRR ≥10% assuming a 60% control-group mortality) will be considered a clinically meaningful “Go” signal, provided that it is accompanied by no clinically important excess in treatment-related serious adverse events and acceptable recruitment and protocol adherence. The 95% CI, including its lower bound, will be reported to characterize statistical uncertainty and the range of effects compatible with the data, but no CI bound will serve as a mandatory success threshold. Point estimates below the 6% threshold will be interpreted together with the 95% CI, safety, secondary outcomes, and feasibility findings rather than being assigned a separate hard “No-Go” boundary. This progression framework is interpretative and non-binding rather than a confirmatory decision rule.

Secondary outcomes are explicitly hypothesis-generating; therefore, no adjustments for multiple comparisons will be made. Given the numerous secondary endpoints, all P-values and 95% CIs for these outcomes are interpreted solely as effect estimates to inform future definitive trials, without controlling for type I error inflation. Single time-point measurements will be summarized descriptively with 95% CIs for group differences where appropriate. For repeatedly measured variables, linear mixed-effects models will describe trajectory changes over time, accounting for within-patient correlation. To address potential skewness in repeated biomarker distributions, log-transformation will be applied prior to modeling, or non-parametric descriptive statistics will be utilized if normality assumptions are violated. To account for potential clustering, site (3 centers) will be incorporated into the mixed models as a fixed effect covariate, which is appropriate given the small number of centers and the exploratory sample size.

Missing data will be handled using multiple imputation as the primary strategy. This decision reflects the nature of the critically ill post-cardiac arrest population, where missingness is often informative and non-random. Multiple imputation by chained equations (MICE) will generate imputed datasets, incorporating baseline characteristics, treatment allocation, and outcome indicators. This approach operates under a Missing at Random (MAR) assumption, conditional on the variables included in the imputation model. The inclusion of comprehensive covariates—including baseline severity scores, treatment assignment, time-dependent physiological parameters, and outcomes assessed prior to dropout—makes the MAR assumption plausible. For longitudinal biomarkers and neurological outcomes, death represents a terminal event that precludes further measurements. In the multiple imputation procedure, measurements occurring after death will not be imputed as if the patient were alive; instead, these missing values will be treated as “missing due to death” and remain missing in the imputed datasets. A pattern-mixture approach, incorporated within the linear mixed-effects models, will explicitly model the trajectory of patients who died as a separate pattern, thereby respecting the informative nature of this missingness. To assess the sensitivity of results to departures from MAR, a tipping-point analysis will be performed to determine the magnitude of departure from MAR that would be required to alter the conclusions. A sensitivity analysis comparing complete-case results with imputed results will further assess robustness.

## Oversight and monitoring

### Composition of the data monitoring committee, its role and reporting structure {28a}

An Executive Committee, comprising the principal investigators from each center and the lead coordinator, will handle day-to-day management. An independent Trial Steering Committee (TSC) of experts in cardiology, emergency medicine, and statistics will provide overall supervision. A dedicated data management team will operate under the Executive Committee. The independent Data Monitoring Committee (DMC), comprising a cardiologist, a neurologist, and a biostatistician, will operate under a formal charter and will conduct unblinded safety reviews after every 15 participants have completed the 30-day follow-up. No formal interim efficacy or futility analyses will be performed. The DMC may recommend trial continuation, modification, temporary suspension, or termination to the steering committee if clinically important safety concerns or an imbalance in treatment-related serious adverse events emerges. All DMC activities and recommendations will be documented and reported to the Trial Steering Committee. All members of the Trial Steering Committee and Data Monitoring Committee will be required to disclose any potential financial or non-financial conflicts of interest before their involvement.

### Interim analyses {28b}

No formal interim analyses for efficacy or futility are planned due to the limited sample size. The independent DMC will conduct safety reviews after every 15 participants have completed the 30-day follow-up and will have exclusive access to unblinded interim safety data. Recommendations regarding trial continuation, modification, temporary suspension, or termination will be based on safety considerations defined in the DMC charter. The final decision to terminate the trial will rest with the steering committee following consultation with the DMC.

### Adverse event reporting and harms

Adverse events (AEs) will be monitored and recorded continuously throughout the trial period. Specific attention will be paid to known levosimendan-associated adverse effects, including hypotension, tachycardia, and arrhythmias. All patients will undergo continuous hemodynamic monitoring (blood pressure, heart rate, cardiac rhythm) during the 24-hour infusion period and for at least 7 days post-infusion.

Hypotension (defined as SBP <90 mmHg or MAP <65 mmHg): Management is stratified by clinical severity. If related to levosimendan, reduce the infusion rate by 50% and consider concomitant vasoactive agents. Discontinue levosimendan immediately if hypotension is refractory to vasoactive support, or if it is accompanied by signs of end-organ hypoperfusion (e.g., altered mental status, oliguria) or hemodynamic instability requiring urgent intervention.

Arrhythmias: Arrhythmia adjudication will be performed by an independent cardiologist at each site, reviewing continuous ECG traces and classified according to AHA guidelines. For supraventricular arrhythmias: eliminate precipitating factors; if drug-related, reduce infusion rate by 50% and administer rate-control medications (e.g., *β*-blockers) to maintain heart rate <130 bpm; discontinue if uncontrolled. For ventricular arrhythmias (including sustained VT/VF): immediately discontinue levosimendan and provide appropriate treatment.

AEs will be categorized as probable, possible, unlikely, or unknown. All serious adverse events (SAEs) will be reported to the principal investigator within 24 hours of occurrence and to the ethics committee within 7 days according to regulatory requirements. Suspected unexpected serious adverse reactions (SUSARs) will be reported immediately to the relevant regulatory authorities. Furthermore, safety data, including mortality, are continuously monitored by the principal investigator and reported to the institutional ethics committee. While formal statistical stopping boundaries for mortality imbalance were not pre-specified in the protocol, any evidence of significant harm will prompt immediate review by the ethics committee, who have the authority to suspend or terminate the trial.

### Frequency and plans for auditing trial conduct {29}

Trial conduct will be subject to regular auditing by an independent GCP monitoring team to ensure compliance with the protocol, GCP guidelines, and regulatory requirements. The monitoring team, which is independent from the study investigators and sponsor, will conduct both on-site and central monitoring throughout the trial period. The frequency of monitoring visits will be risk-based, with an initial full audit followed by quarterly reviews, supplemented by additional triggered audits if protocol deviations or data quality issues are identified. All audit findings will be reported directly to the independent Data Monitoring Committee and the institutional review board. The principal investigator will be responsible for implementing corrective and preventive actions in response to audit recommendations.

This study protocol has been written in accordance with the Standard Protocol Items: Recommendations for Interventional Trials (SPIRIT) guidelines. The completed SPIRIT checklist is provided as Supplementary Table S1.

## Protocol management and ethics

### Plans for communicating important protocol amendments to relevant parties {31}

Any protocol amendments will be first submitted for approval to the institutional review board and relevant regulatory authorities. Substantial modifications (e.g., changes to eligibility criteria, outcomes, interventions, or analytical methods) will also be communicated to all investigators and updated in the clinical trial registry (ClinicalTrials.gov NCT05956431) within 21 days of approval. Minor administrative changes will be documented and maintained in the trial master file. All approved amendments will be implemented consistently across all participating sites, and relevant training will be provided to ensure protocol adherence. Trial participants will be informed of any modifications that might affect their participation or welfare.

### Who will take informed consent? {32a}

The trial will be conducted in accordance with the Declaration of Helsinki and comply with China's Regulations on Ethical Review of Biomedical Research Involving Humans (Article 44) and Good Clinical Practice guidelines for emergency research involving temporarily incapacitated subjects. Since subjects lack decision-making capacity post-ROSC, proxy consent must be provided by the legal surrogate (prioritized as spouse > parents > adult children per Civil Code). Paper-based consent forms will be completed with handwritten signatures, supplemented by electronic archiving in accordance with China's Electronic Signature Law.

### Additional consent provisions for collection and use of participant data and biological specimens {32b}

The informed consent form, signed by the legally authorized representative, includes specific provisions for the collection and storage of biological specimens for use in future ancillary studies that have been approved by the institutional review board. Approval for each specific ancillary study will be obtained from the IRB prior to the use of the samples or data.

### Confidentiality {33}

Confidentiality of all participant data will be strictly maintained in accordance with Chinese regulatory requirements and international standards. Personal identifiable information is collected minimally and stored separately from study data under strict access controls. All electronic data are stored on secure, encrypted servers with regular backups. Analyses and publications will utilize only fully de-identified data. Access to identifiable information is restricted to authorized personnel exclusively for study purposes. Study data will be retained for a minimum of 15 years after study completion as mandated.

### Provisions for post-trial care {34}

The trial does not provide specific post-trial care beyond standard clinical care provided by the treating institutions. Any medical care required due to complications potentially related to the study intervention (levosimendan or procedures) will be managed as per the hospital's standard practices. Participants retain all legal rights.

## Discussion

Clinical evidence for levosimendan in related low-output and critical-care settings remains heterogeneous and inconclusive (28–32). Studies in cardiogenic shock, extracorporeal membrane oxygenation weaning, and cardiac-surgery populations differ substantially in patient phenotype, comparator therapy, timing of drug administration, and baseline ventricular function, which limits direct extrapolation to PAMD after OHCA (28–32). These evidence gaps support the need for a population-specific randomized trial in post-cardiac-arrest myocardial dysfunction. Our protocol addresses this gap by initiating the study infusion within 6 hours after ROSC, targeting patients with confirmed LVEF <40%, and restricting enrollment to those achieving ROSC within 60 minutes, thereby aligning treatment timing with the anticipated early course of myocardial dysfunction and improving cohort homogeneity.

During the trial, all inotropes other than the study drug are prohibited in both arms. This restriction reflects the absence of robust PAMD-specific evidence that routine inotropic therapy improves survival. Current post-cardiac-arrest guidance addresses hemodynamic support, while evidence concerning mortality effects of routinely used vasopressors and inotropes in acute myocardial infarction-related cardiogenic shock is low quality and heterogeneous and does not establish a survival benefit (33, 34). Given the uncertain risk–benefit balance, clinical equipoise exists for this protocol, with protocol-defined open-label rescue available for refractory tissue hypoperfusion.

The rationale for testing levosimendan administration within 6 hours post-ROSC is based on the pathophysiological premise that this timing would allow the drug to reach therapeutic levels coinciding with the anticipated nadir of post-arrest myocardial dysfunction. This strategy constitutes the central hypothesis of our trial. Pharmacokinetic studies (35–37) indicate that levosimendan requires approximately 1 hour to achieve therapeutic plasma levels and 4-5 hours to reach steady state. This onset profile is critical because clinical data from Laurent et al. (38) demonstrate that hemodynamic decline begins early; in their study of 148 patients, nearly half (49%) developed significant impairment (cardiac index <2.2 L/min/m^2^ and mean arterial pressure <75 mmHg) within the first 6 hours post-ROSC, with the cardiac index reaching its lowest point at 8 hours. Initiating infusion by the 6-hour mark ensures that therapeutic drug concentrations are present as cardiac function approaches this vulnerable nadir. The rationale for early administration is also informed by the exploratory timing analysis of Rysz et al. (23), in which initiation within 0–6 hours after ICU admission was associated with lower adjusted 30-day mortality than later initiation. Because the study was observational and susceptible to residual confounding, this finding is hypothesis-generating rather than confirmatory.

Witnessed arrest and shorter resuscitation duration are associated with better outcomes after OHCA (39–41). In particular, 30-day survival decreases as CPR-to-ROSC time increases, although meaningful survival remains possible after prolonged resuscitation (41). The <60-minute eligibility cutoff is therefore a pragmatic threshold intended to limit extreme heterogeneity in global ischemic burden and preserve a plausible opportunity for neurological and myocardial recovery; it should not be interpreted as a validated boundary of biological reversibility.

For patients with low cardiac output syndrome after ROSC (LVEF <40%), a meta-analysis by Harrison et al. (42) (14 RCTs, *n* = 1,155 cardiac surgery patients) demonstrated that levosimendan significantly reduces postoperative mortality, dialysis requirement, and atrial fibrillation incidence (*P* < 0.05). Crucially, benefits were confined to patients with LVEF <40% (risk difference −7.0%; 95% CI −11.0% to −3.1%; *P* < 0.001), with no significant effect in preserved EF subgroups (+1.1%; −3.8% to +5.9%; *P* = 0.66). Therefore, an LVEF <40% threshold was selected to target responders to levosimendan's calcium-sensitizing mechanisms, ensure cohort homogeneity, and enhance clinical relevance. A recent GRADE-assessed systematic review comparing levosimendan with milrinone in cardiac-surgery patients found no clear superiority across key hemodynamic and clinical outcomes, highlighting persistent uncertainty regarding comparative inotropic strategies and supporting the need for population-specific randomized evidence (43).

## Trial status

Protocol: v2.0 of 2 June 2023.

Recruitment was originally planned to commence on 1 August 2023 following trial registration and ethics approval; however, logistical delays in funding allocation, investigational-drug availability, and sequential ethics requirements at the sub-centers postponed active screening until May 2026. As of this manuscript revision, four patients had been screened at the coordinating center, one met the eligibility criteria but declined participation, and no participant had been randomized. The active recruitment period has therefore been extended through December 2027, subject to applicable ethics approval and trial-registry updating. No trial outcome data are reported in this protocol article.

## Data Availability

The full protocol, de-identified participant-level dataset, data dictionary, and statistical code will be made available upon reasonable request to qualified researchers beginning 12 months after completion of the trial. Requests require approval from the study's Steering Committee and must include a methodologically sound proposal with approval from an institutional review board. Data will be shared under a data use agreement that prohibits re-identification and ensures appropriate use consistent with participant informed consent.

## Supplementary material

Supplementary Table S1 SPIRIT 2025 checklist of items to address in a randomized trial protocol.

## Glossary

AE: adverse event
BUN: blood urea nitrogen
CA: cardiac arrest
CI: confidence interval
CPC: cerebral performance category
Cr: creatinine
CrCl: creatinine clearance
DMC: Data Monitoring Committee
eCRF: electronic case report form
ECPR: extracorporeal cardiopulmonary resuscitation
ECG: electrocardiogram
eCrCl: estimated creatinine clearance
ED: emergency department
EF: ejection fraction
EMS: emergency medical services
ERK: extracellular signal-regulated kinase
FAS: full analysis set
GCP: good clinical practice
GCS: Glasgow Coma Scale
GWR: gray-white matter ratio
ICU: intensive care unit
IL-6: interleukin-6
IRI: ischemia-reperfusion injury
IRB: Institutional Review Board
ITT: intention-to-treat
KATP: ATP-sensitive potassium channel
LAR: legally authorized representative
LVEF: left ventricular ejection fraction
MAP: mean arterial pressure
mRS: modified Rankin Scale
NGAL: neutrophil gelatinase-associated lipocalin
NMPA: National Medical Products Administration
NSE: neuron-specific enolase
NT-proBNP: N-terminal pro-B-type natriuretic peptide
OHCA: out-of-hospital cardiac arrest
OR: odds ratio
PAMD: post-cardiac arrest myocardial dysfunction
PCAS: post-cardiac arrest syndrome
PiCCO: pulse index continuous cardiac output
PI: principal investigator
PPS: per-protocol set
RCT: randomized controlled trial
REDCap: research electronic data capture
ROS: reactive oxygen species
ROSC: return of spontaneous circulation
RRT: renal replacement therapy
SAE: serious adverse event
SAP: statistical analysis plan
SAS: statistical analysis system
SBP: systolic blood pressure
SD: standard deviation
SIRS: systemic inflammatory response syndrome
SOP: standard operating procedure
SPIRIT: Standard Protocol Items: Recommendations for Interventional Trials
SUSAR: suspected unexpected serious adverse reaction
TNF-*α*: tumor necrosis factor-alpha
TTE: transthoracic echocardiography
VF: ventricular fibrillation
VT: ventricular tachycardia
