# Supplementary material for: Early use of levosimendan to improve survival and neurological outcomes after cardiac arrest with myocardial dysfunction: a protocol for a randomized controlled trial
Source: Front Cardiovasc Med. 2026 Sep 17;13:1850394. doi: 10.3389/fcvm.2026.1850394 (PMC13627038; doi:10.3389/fcvm.2026.1850394)
Supplement: Supplementary file 1 [file Table1.docx]

**Supplementary Table S1. SPIRIT 2025 checklist of items to address in a randomized trial protocol***

Locations are reported by manuscript section/subsection headings rather than page numbers, because pagination may change during production.

| **Section / Topic** | **No** | **SPIRIT 2025 checklist item description** | **Location in manuscript** |
| --- | --- | --- | --- |
| **Administrative information** | | |  |
| Title and structured summary | 1a | Title stating the trial design, population, and interventions, with identification as a protocol | Title |
|  | 1b | Structured summary of trial design and methods, including items from the World Health Organization Trial Registration Data Set | Abstract |
| Protocol version | 2 | Version date and identifier | Trial status – Protocol |
| Roles and responsibilities | 3a | Names, affiliations, and roles of protocol contributors | Author list and affiliations; Author contributions; Generative AI statement |
|  | 3b | Name and contact information for the trial sponsor | Methods: patient and public involvement, trial design – Trial design (trial sponsor and contact) |
|  | 3c | Role of trial sponsor and funders in design, conduct, analysis, and reporting of trial; including any authority over these activities | Methods: patient and public involvement, trial design – Trial design (role of sponsor and funders); Funding |
|  | 3d | Composition, roles, and responsibilities of the coordinating site, steering committee, endpoint adjudication committee, data management team, and other individuals or groups overseeing the trial, if applicable | Oversight and monitoring; Oversight and monitoring – Composition of the data monitoring committee, its role and reporting structure |
| **Open science** | | |  |
| Trial registration | 4 | Name of trial registry, identifying number (with URL), and date of registration. If not yet registered, name of intended registry | Abstract – Clinical Trial Registration (registry, registration number, URL, and registration date) |
| Protocol and statistical analysis plan | 5 | Where the trial protocol and statistical analysis plan can be accessed | Open science – Where the trial protocol and statistical analysis plan can be accessed |
| Data sharing | 6 | Where and how the individual de-identified participant data (including data dictionary), statistical code, and any other materials will be accessible | Data availability statement |
| Funding and conflicts of interest | 7a | Sources of funding and other support (e.g., supply of drugs) | Funding |
|  | 7b | Financial and other conflicts of interest for principal investigators and steering committee members | Oversight and monitoring – Composition of the data monitoring committee, its role and reporting structure; Conflict of interest |
| Dissemination policy | 8 | Plans to communicate trial results to participants, healthcare professionals, the public, and other relevant groups (e.g., reporting in trial registry, plain language summary, publication) | Open science – Plans to communicate trial results |
| **Introduction** | | |  |
| Background and rationale | 9a | Scientific background and rationale, including summary of relevant studies (published and unpublished) examining benefits and harms for each intervention | Introduction – Background and rationale; Discussion |
|  | 9b | Explanation for choice of comparator | Introduction – Explanation for the choice of comparators |
| Objectives | 10 | Specific objectives related to benefits and harms | Objectives – Primary objectives; Secondary objectives |
| **Methods: Patient and public involvement, trial design** | | |  |
| Patient and public involvement | 11 | Details of, or plans for, patient or public involvement in the design, conduct, and reporting of the trial | Methods: patient and public involvement, trial design – Patient and public involvement |
| Trial design | 12 | Description of trial design including type of trial (e.g., parallel group, crossover), allocation ratio, and framework (e.g., superiority, equivalence, non-inferiority, exploratory) | Methods: patient and public involvement, trial design – Trial design |
| **Methods: Participants, interventions, and outcomes** | | |  |
| Trial setting | 13 | Settings (e.g., community, hospital) and locations (e.g., countries, sites) where the trial will be conducted | Participants, interventions, and outcomes – Study setting |
| Eligibility criteria | 14a | Eligibility criteria for participants | Participants, interventions, and outcomes – Eligibility criteria |
|  | 14b | If applicable, eligibility criteria for sites and for individuals who will deliver the interventions (e.g., surgeons, physiotherapists) | Participants, interventions, and outcomes – Eligibility criteria for sites and for individuals who will deliver the interventions |
| Intervention and comparator | 15a | Intervention and comparator with sufficient details to allow replication including how, when, and by whom they will be administered. If relevant, where additional materials describing the intervention and comparator (e.g., intervention manual) can be accessed | Participants, interventions, and outcomes – Interventions – Intervention description |
|  | 15b | Criteria for discontinuing or modifying allocated intervention/comparator for a trial participant (e.g., drug dose change in response to harms, participant request, or improving/worsening disease) | Participants, interventions, and outcomes – Criteria for discontinuing or modifying allocated interventions |
|  | 15c | Strategies to improve adherence to intervention/comparator protocols, if applicable, and any procedures for monitoring adherence (e.g., drug tablet return, sessions attended) | Participants, interventions, and outcomes – Strategies to improve adherence to interventions |
|  | 15d | Concomitant care that is permitted or prohibited during the trial | Participants, interventions, and outcomes – Relevant concomitant care permitted or prohibited during the trial |
| Outcomes | 16 | Primary and secondary outcomes, including the specific measurement variable (e.g., systolic blood pressure), analysis metric (e.g., change from baseline, final value, time to event), method of aggregation (e.g., median, proportion), and time point for each outcome | Participants, interventions, and outcomes – Outcomes (Mortality at 30 days; Secondary outcomes; Safety) |
| Harms | 17 | How harms are defined and will be assessed (e.g., systematically, non-systematically) | Participants, interventions, and outcomes – Harms; Oversight and monitoring – Adverse event reporting and harms |
| Participant timeline | 18 | Time schedule of enrollment, interventions (including any run-ins and washouts), assessments, and visits for participants. A schematic diagram is highly recommended (see Figure) | Participants, interventions, and outcomes – Outcomes; Figure 1; Table 1 |
| Sample size | 19 | How sample size was determined, including all assumptions supporting the sample size calculation | Participants, interventions, and outcomes – Sample size |
| Recruitment | 20 | Strategies for achieving adequate participant enrollment to reach target sample size | Participants, interventions, and outcomes – Recruitment |
| **Methods: Assignment of interventions** | | |  |
| Randomization: |  |  |  |
| Sequence generation | 21a | Who will generate the random allocation sequence and the method used | Assignment of interventions: allocation – Sequence generation |
|  | 21b | Type of randomization (simple or restricted) and details of any factors for stratification. To reduce predictability of a random sequence, other details of any planned restriction (e.g., blocking) should be provided in a separate document that is unavailable to those who enroll participants or assign interventions | Assignment of interventions: allocation – Sequence generation |
| Allocation concealment  mechanism | 22 | Mechanism used to implement the random allocation sequence (e.g., central computer/telephone; sequentially numbered, opaque, sealed containers), describing any steps to conceal the sequence until interventions are assigned | Assignment of interventions: allocation – Concealment mechanism |
| Implementation | 23 | Whether the personnel who will enroll and those who will assign participants to the interventions will have access to the random allocation sequence | Assignment of interventions: allocation – Implementation |
| Blinding | 24a | Who will be blinded after assignment to interventions (e.g., participants, care providers, outcome assessors, data analysts) | Assignment of interventions: blinding – Who will be blinded |
|  | 24b | If blinded, how blinding will be achieved and description of the similarity of interventions | Assignment of interventions: blinding – How blinding will be achieved |
|  | 24c | If blinded, circumstances under which unblinding is permissible, and procedure for revealing a participant’s allocated intervention during the trial | Assignment of interventions: blinding – Procedure for unblinding if needed |
| **Methods: Data collection, management, and analysis** | | |  |
| Data collection methods | 25a | Plans for assessment and collection of trial data, including any related processes to promote data quality (e.g., duplicate measurements, training of assessors) and a description of trial instruments (e.g., questionnaires, laboratory tests) along with their reliability and validity, if known. Reference to where data collection forms can be accessed, if not in the protocol | Data collection and management – Plans for assessment and collection of outcomes |
|  | 25b | Plans to promote participant retention and complete follow-up, including list of any outcome data to be collected for participants who discontinue or deviate from intervention protocols | Data collection and management – Plans to promote participant retention and complete follow-up |
| Data management | 26 | Plans for data entry, coding, security, and storage, including any related processes to promote data quality (e.g., double data entry; range checks for data values). Reference to where details of data management procedures can be accessed, if not in the protocol | Data collection and management – Data management |
| Statistical methods | 27a | Statistical methods used to compare groups for primary and secondary outcomes, including harms | Statistical methods – Statistical methods for primary and secondary outcomes |
|  | 27b | Definition of who will be included in each analysis (e.g., all randomized participants), and in which group | Statistical methods – Statistical methods for primary and secondary outcomes |
|  | 27c | How missing data will be handled in the analysis | Statistical methods – Statistical methods for primary and secondary outcomes (missing data) |
|  | 27d | Methods for any additional analyses (e.g., subgroup and sensitivity analyses) | Statistical methods – Statistical methods for primary and secondary outcomes (additional and sensitivity analyses) |
| **Methods: Monitoring** | | |  |
| Data monitoring committee | 28a | Composition of data monitoring committee (DMC); summary of its role and reporting structure; statement of whether it is independent from the sponsor and funder; conflicts of interest and reference to where further details about its charter can be found, if not in the protocol. Alternatively, an explanation of why a DMC is not needed | Oversight and monitoring – Composition of the data monitoring committee, its role and reporting structure |
|  | 28b | Explanation of any interim analyses and stopping guidelines, including who will have access to these interim results and make the final decision to terminate the trial | Oversight and monitoring – Interim analyses |
| Trial monitoring | 29 | Frequency and procedures for monitoring trial conduct. If there is no monitoring, give explanation | Oversight and monitoring – Frequency and plans for auditing trial conduct |
| **Ethics** | | |  |
| Research ethics approval | 30 | Plans for seeking research ethics committee/institutional review board approval | Methods: patient and public involvement, trial design – Trial design; Ethics statement |
| Protocol amendments | 31 | Plans for communicating important protocol modifications to relevant parties | Protocol management and ethics – Plans for communicating important protocol amendments to relevant parties |
| Consent or assent | 32a | Who will obtain informed consent or assent from potential trial participants or authorized proxies, and how | Protocol management and ethics – Who will take informed consent?; Ethics statement |
|  | 32b | Additional consent provisions for collection and use of participant data and biological specimens in ancillary studies, if applicable | Protocol management and ethics – Additional consent provisions for collection and use of participant data and biological specimens |
| Confidentiality | 33 | How personal information about potential and enrolled participants will be collected, shared, and maintained in order to protect confidentiality before, during, and after the trial | Protocol management and ethics – Confidentiality |
| Ancillary and post-trial care | 34 | Provisions, if any, for ancillary and post-trial care, and for compensation to those who suffer harm from trial participation | Protocol management and ethics – Provisions for post-trial care |

*We strongly recommend reading this checklist in conjunction with the SPIRIT 2025 Explanation and Elaboration and the SPIRIT 2025 Expanded Checklist for important clarifications on all the items. We also recommend reading relevant SPIRIT extensions. See [www.consort-spirit.org](http://www.consort-spirit.org)

Citation: Chan A-W, Boutron I, Hopewell S, Moher D, Schulz KF, Collins GS, et al. SPIRIT 2025 statement: updated guideline for protocols of randomised trials. BMJ 2025;389:e081477. <https://dx.doi.org/10.1136/bmj-2024-081477>

© 2025 Chan A-W et al. This is an Open Access article distributed under the terms of the Creative Commons Attribution License (<https://creativecommons.org/licenses/by/4.0/>), which permits unrestricted use, distribution, and reproduction in any medium, provided the original work is properly cited.
